# Epstein-Barr virus-encoded EBNA1 enhances RNA polymerase III-dependent EBER expression through induction of EBER-associated cellular transcription factors

**DOI:** 10.1186/1476-4598-9-241

**Published:** 2010-09-15

**Authors:** Thomas J Owen, John D O'Neil, Christopher W Dawson, Chunfang Hu, Xiaoyi Chen, Yunhong Yao, Victoria HJ Wood, Louise E Mitchell, Robert J White, Lawrence S Young, John R Arrand

**Affiliations:** 1 School of Cancer Sciences, University of Birmingham, Birmingham B15 2TT, UK; 2 Dept. of Pathology, Guangdong Medical College, Zhanjiang, Guangdong, China; 3 Beatson Institute for Cancer Research, Switchback Road, Glasgow G61 1BD, UK

## Abstract

**Background:**

Epstein-Barr Virus (EBV)-encoded RNAs (EBERs) are non-polyadenylated RNA molecules transcribed from the EBV genome by RNA polymerase III (pol III). EBERs are the most abundant viral latent gene products, although the precise mechanisms by which EBV is able to achieve such high levels of EBER expression are not fully understood. Previously EBV has been demonstrated to induce transcription factors associated with EBER expression, including pol III transcription factors and ATF-2. We have recently demonstrated that EBV-encoded nuclear antigen-1 (EBNA1) induces cellular transcription factors, and given these findings, we investigated the role of EBNA1 in induction of EBER-associated transcription factors.

**Results:**

Our data confirm that in epithelial cells EBNA1 can enhance cellular pol III transcription. Transient expression of EBNA1 in Ad/AH cells stably expressing the EBERs led to induction of both EBER1 and EBER2 and conversely, expression of a dominant negative EBNA1 led to reduced EBER expression in EBV-infected Ad/AH cells. EBNA1 can induce transcription factors used by EBER genes, including TFIIIC, ATF-2 and c-Myc. A variant chromatin precipitation procedure showed that EBNA1 is associated with the promoters of these genes but not with the promoters of pol III-transcribed genes, including the EBERs themselves. Using shRNA knock-down, we confirm the significance of both ATF-2 and c-Myc in EBER expression. Further, functional induction of a c-Myc fusion protein led to increased EBER expression, providing c-Myc binding sites upstream of EBER1 were intact. *In vivo *studies confirm elevated levels of the 102 kD subunit of TFIIIC in the tumour cells of EBV-positive nasopharyngeal carcinoma biopsies.

**Conclusions:**

Our findings reveal that EBNA1 is able to enhance EBER expression through induction of cellular transcription factors and add to the repertoire of EBNA1's transcription-regulatory properties.

## Background

In the 46 years since its discovery, the ubiquitous Epstein-Barr Virus (EBV) has been found to be closely associated with a wide range of epithelial and lymphoid malignancies [1]. During all typical forms of latent EBV infection and B-cell immortalisation, two highly expressed gene products are the non-polyadenylated RNAs, Epstein-Barr Virus encoded RNAs 1 and 2 (EBER1 and EBER2). Although no definitive role for the EBERs in cell transformation or malignant growth has been elucidated, several studies highlight the EBERs as making a significant contribution to EBV-associated malignancies [2-5] probably through direct interactions with cellular proteins with which they are known to form complexes. These include PKR [6,7], RIG-I [8], La [9] and ribosomal protein L22 [10-12]. In addition, the EBERs can induce a number of cytokines in lymphocytes [13,14] and insulin-like growth factor 1 in epithelial cells [15,16]. Induction of these autocrine growth factors hints at the transforming role the EBERs may play in EBV pathology. Indeed, stable expression of EBERs in immortalised nasopharyngeal epithelial cells confers resistance to apoptotic stress [17].

EBER1 and EBER2 are transcribed from the EBV genome by RNA polymerase III (pol III) and as such contain elements (intragenic A and B boxes) typical of those required for pol III transcription [18,19]. Interestingly, both EBER genes also possess upstream transcriptional regulatory regions that are more typical of pol II promoters; ATF, Sp1 and TATA binding sites [20]. In addition, c-Myc has been shown to bind *in vitro *and *in vivo *to two E-Boxes upstream of the EBER1 gene and, in the context of a minimal promoter, the isolated Myc-binding locus was transcriptionally active in the presence of c-Myc [21].

Several oncogenic viruses including hepatitis B virus, human T-cell leukaemia virus type 1, adenovirus, polyoma and SV40, stimulate RNA polymerase III-mediated transcription by the induction of increased levels of pol III-specific transcription factors [22-26] and a number of trans-acting factors are known to be important in the transcriptional control of the EBERs [2,9,27,28]. Recently, EBV has been shown to induce the cellular transcription factors TFIIIB and TFIIIC (leading to induction of general pol III-mediated transcription) and the typical pol II transcription factor ATF-2, that enhance expression of EBER1 and EBER2 [29].

Following EBV infection of B-lymphocytes, temporally the EBERs are the last EBV latent gene product to be expressed [28,30]. The aforementioned observations make it tempting to propose that another EBV latent gene product may be involved in the regulation of EBER expression through the induction of EBER-specific transcription factors. Indeed, evidence of such a phenomenon is found in our previous study which demonstrated the ability of EBNA1 to induce both the transcription of the genes and activity of the proteins of members of the AP-1 family, including ATF-2 [31]. EBNA1 is also known to have transcriptional regulatory properties on the viral Cp, Qp and LMP1 promoters [32-34].

Although the precise mechanisms by which EBERs may function in oncogenesis are not yet resolved, it is clear that they are an important component of the EBV latent gene complement. In this study, we aim to pinpoint the mechanisms by which EBV is able to increase EBER expression through induction of transcriptional components. Specifically, we test the hypothesis that EBNA1 may be the latent gene product involved in the regulation of both classic pol II and pol III transcription factors associated with EBER expression. Our results confirm the hypothesis and define additional EBNA1-mediated mechanisms of transcriptional regulation.

## Results

### EBNA1 induces multiple TFIIIC subunits

**Figure 1:**
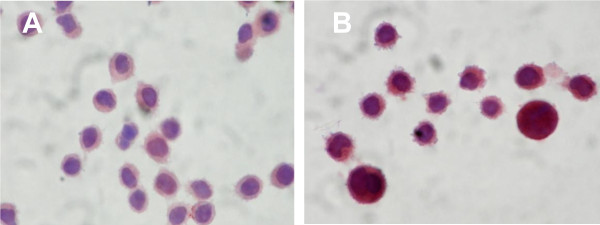
**Induction of TFIIIC102 by EBNA1**. Immunohistochemical staining for TFIIIC-102 in (A) Ad/AH-Neo and (B) Ad/AH-EBNA1-cl3 cells.

**Figure 2:**
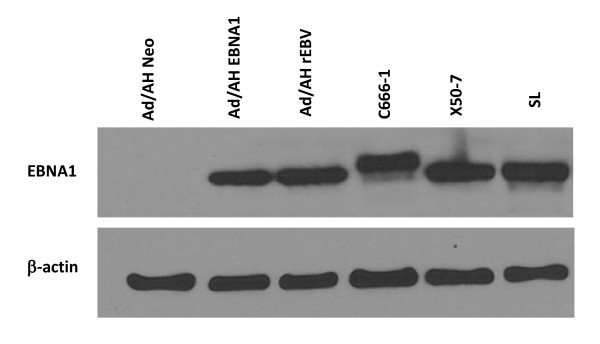
**EBNA1 is expressed at physiological levels in Ad/AH-EBNA1-cl8 cells**. Immunoblot analysis of EBNA1 expression in the epithelial cell lines Ad/AH, Ad/AH-EBNA1-cl8, Ad/AH-EBV, the EBV-positive NPC line C666-1 and the lymphoblastoid cell lines X50-7 and SL (B-cells immortalised with the B95-8 strain of EBV). Note the larger size of C666-1 EBNA1, presumably due to a larger Gly-Ala repeat in this strain of EBV. β-actin served as a loading control.

EBV has previously been shown to induce RNA polymerase III activity via TFIIIC expression in both lymphoid and epithelial cells [29], although the viral gene product responsible for this induction remained unidentified. Affymetrix array analysis revealed a 2.6-fold induction of RNA encoding the 102kd subunit of TFIIIC (TFIIIC102) in response to EBNA1 [35], indicating that this EBV latent gene product may be responsible for inducing TFIIIC expression. To confirm this observation, immunohistochemical staining for TFIIIC102 was conducted and revealed a clear induction of TFIIIC102 in cells expressing EBNA1 (Figure 1). However, the Ad/AH-EBNA1-cl3 cells that were used in these experiments overexpressed EBNA1 [35]. In order to ensure that the observed effects of EBNA were not due to non-physiological levels of expression, another Ad/AH cell line, Ad/AH-EBNA1-cl8, that stably expresses EBNA1 at levels comparable to those seen in EBV latently infected cells (Figure 2) was used in all the following experiments and is referred to as Ad/AH-EBNA1. The EBNA1 derivatives of AGS and Hone-1 cells were previously shown to express physiological levels of EBNA1 [35].

**Figure 3:**
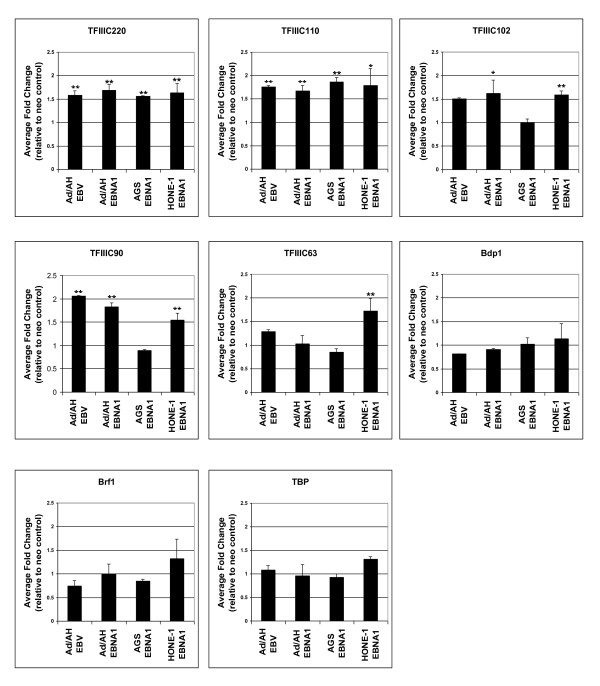
**EBNA1 induces TFIIIC genes, but not TFIIIB transcripts in Ad/AH, AGS, and HONE-1 cells**. Levels of transcripts were assayed using RT-qPCR in epithelial cell lines expressing neomycin control plasmids or EBNA1. For comparative purposes, EBV-infected Ad/AH cells are included. Fold change values are between neo control cells and EBNA1-expressing or EBV-infected cells, as appropriate. All data are means of three biological replicates analysed in technical triplicate. Error bars represent the standard deviation in fold change between biological replicates. Asterisks indicate that the fold change between neo control and EBNA1-transfected or EBV-positive cells is statistically significant. *, P ≤ 0.05; **, P ≤ 0.01.

To ascertain EBNA1's capability to induce multiple TFIIIC subunits, Q-PCR was carried out for all 5 TFIIIC subunits that have been shown to respond to EBV [29]. In order to compare directly the effect of EBNA1 and EBV on TFIII induction, Ad/AH-EBV cells were included in this analysis. Transcripts encoding TFIIIC subunits were induced in EBNA1-expressing derivatives of 3 distinct carcinoma cell models, Ad/AH, AGS and HONE-1 (Figure 3). As observed previously [29], the pattern of subunit mRNAs induced varied between cell types, but in each case EBNA1 induced a statistically significant enhancement of the expression of at least two TFIIIC subunits. The effect was most pronounced in Ad/AH and HONE-1 cells. In contrast, there was no significant increase in mRNAs encoding the TFIIIB subunits Bdp1, Brf1 and TBP (Figure 3). In all cases the effect of EBNA1 in Ad/AH cells essentially mirrored that of EBV. We conclude that EBNA1 can have a selective effect on expression of TFIIIC.

**Figure 4:**
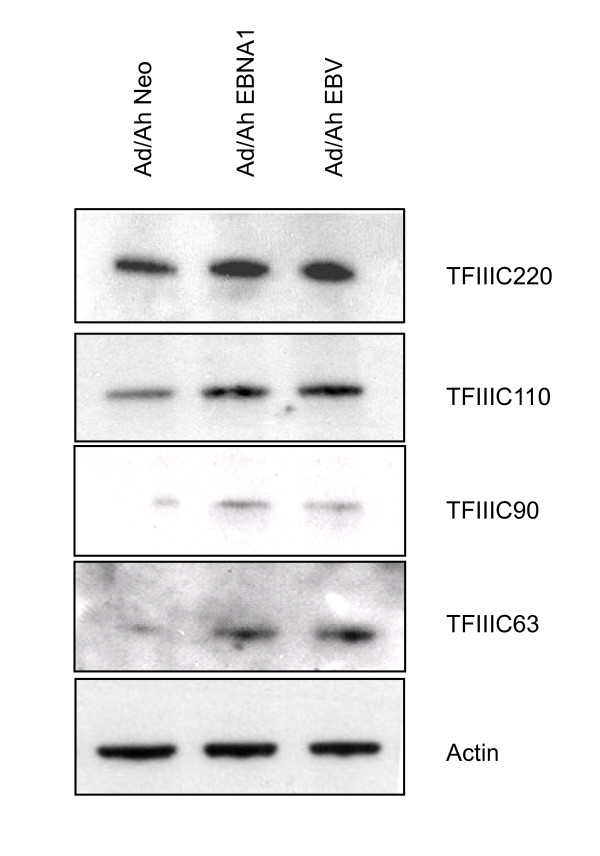
**EBNA1 induces TFIIIC proteins in Ad/AH cells**. Immunoblotting for expression of TFIIIC subunits in protein extracts from Ad/AH cells expressing neomycin control plasmids or EBNA1, and in Ad/AH cells infected with EBV.

To verify that the transcriptional enhancement resulted in increased protein expression the levels of TFIIIC subunits expressed in Ad/AH, Ad/AH-EBNA1 and Ad/AH-EBV cells were examined by immunoblotting (Figure 4). The levels of 4 of the 5 subunits were found to be induced by EBNA1 to levels equivalent to those seen following EBV infection. For technical reasons we were unable to confirm the expression of TFIIIC-102. However, in view of the fact that translation of the other 4 subunits reflects the induction of the mRNA and that immunohistochemistry (Figure 1) shows induction of TFIIIC-102 in response to EBNA1, we conclude that TFIIIC-102 protein is upregulated and, like EBV, EBNA1 induces the expression of all 5 TFIIIC subunits in Ad/AH cells.

### EBNA1 increases general pol III-mediated transcription

**Figure 5:**
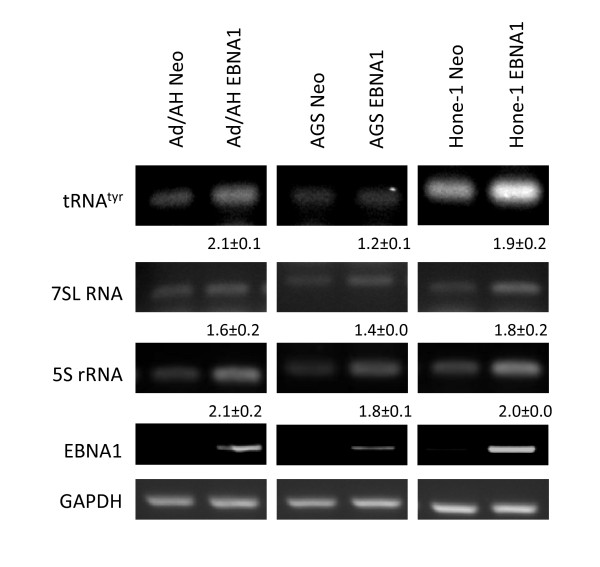
**EBNA1 induces cellular pol III genes in Ad/AH, AGS, and HONE-1 cells**. RT-PCR analysis of the levels of cellular polIII-transcribed genes tRNA^tyr^, 5S rRNA and 7SL RNA in epithelial cell lines expressing neomycin control plasmids or EBNA1. Band intensities from experiments conducted in biological triplicate were quantitated using densitometry and the mean fold change (± standard deviation (SD)) between EBNA1-positive cells and the corresponding control is indicated under the appropriate band.

EBV has previously been shown to increase the expression of several pol III targets [29,36,37]. To determine whether this could be due to the expression of EBNA1, RT-PCR for a number of cellular pol III-transcribed genes was carried out across the panel of EBNA1-expressing epithelial cells. Expression of EBNA1 in all cell lines resulted in increases in expression of the cellular pol III-transcribed genes tRNA^Tyr^, 7SL RNA and 5S rRNA (Figure 5), similar to those seen in EBV-infected epithelial cells. Therefore, EBNA1 may be responsible, at least in part, for the increase in expression of endogenous pol III products that is induced by EBV. Although the induction is quantitatively modest, a comparable level of tRNA overexpression has been shown to be capable of proliferative and oncogenic effects in immortalised mouse embryonic fibroblasts [38].

### EBNA1 induces activated ATF-2

**Figure 6:**
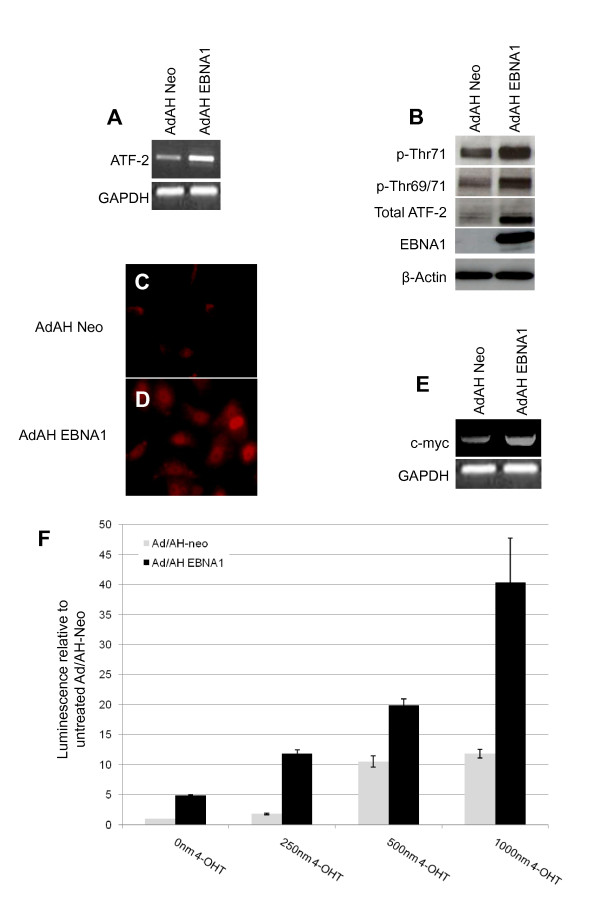
**EBNA1 induces ATF-2 and c-Myc in Ad/AH cells**. (a) RT-PCR analysis shows that transcription of ATF-2 is increased in EBNA1-expressing cells. (b) Western blot analysis of total, mono- (Thr71) and dual-phosphorylated (Thr69/71) ATF-2 levels in Ad/AH-Neo and -EBNA1 cells. (c) and (d) IF staining for total ATF-2. (e) RT-PCR analysis shows that transcription of c-myc is increased in EBNA1-expressing cells. (f) Luciferase reporter assay following transient transfection of Ad/AH cells stably expressing c-MycER and either EBNA1 or an empty vector control plasmid (Neo) with a c-Myc luciferase reporter plasmid (pX-CMVp-Luc) with treatment of cells with 0 nM, 250 nM, 500 nM or 1000 nM 4-Hydroxytamoxifen for 24 hours.

In addition to the influence of pol III-specific transcription factors, EBER expression is reliant upon factors more typically associated with pol II transcription, such as ATF-2 which has been shown to be increased in EBV-infected cells [29]. ATF-2 was also found to bind and activate the promoter of the gene encoding TFIIIC220 [29]. Consistent with our previous study [31], a robust increase in levels of ATF-2 mRNA was observed in EBNA1-expressing Ad/AH cells (Figure 6a), along with an increase in the total, mono- and dual-phosphorylated forms of the protein (Figure 6b, 6c, 6d).

### EBNA1 increases c-Myc expression and activity

c-Myc has a binding site upstream of the EBER genes [21] and has been shown to be transcriptionally induced in EBNA1-expressing Ad/AH cells [35]. This upregulation was confirmed by RT-PCR analysis (Figure 6e). To assess whether the enhanced expression was associated with an increase in the transcriptional activation capacity of c-Myc, Ad/AH-Neo and -EBNA1 cells stably expressing a functionally inducible c-Myc fusion protein (c-MycER) were assessed for c-Myc-mediated activation of a reporter construct following functional induction of the c-Myc fusion protein with 4-hydroxytamoxifen (4-OHT) (Figure 6f). At all tamoxifen concentrations, enhancement of c-Myc function was observed in both Neo- and EBNA1-expressing cells, with activity being around 4- to 5-fold higher in EBNA1-expressing cells compared with their Neo counterparts. This is consistent with there being an increased transcription-enhancing activity of c-Myc in the presence of EBNA1. In both Ad/AH-Neo and -EBNA1 cells, the increase of c-Myc activity in response to 4-OHT treatment was titratable, with increased 4-OHT concentrations resulting in increased levels of c-Myc-induced transcriptional activation.

### EBNA1 is present at the promoters of induced, EBER-associated transcription factor genes but not at the promoters of pol III-transcribed genes

**Figure 7:**
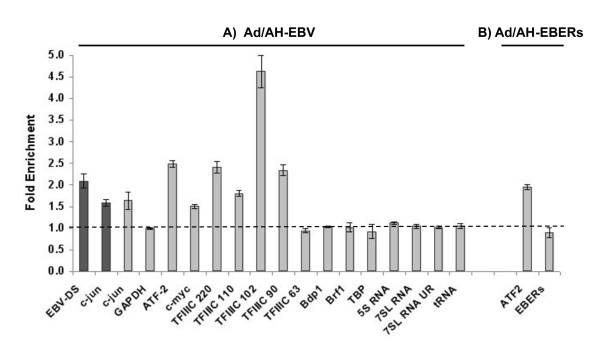
**EBNA1 is present at the promoters of induced, EBER-associated transcription factor genes but not at the promoters of pol III transcribed genes**. PCR for target promoter region DNA was performed for multiple genes on cross-linked genomic DNA pulled down by HaloEBNA1 fusion protein or, as control, when pull down of HaloEBNA1 and associated cross-linked DNA was specifically blocked by a blocking ligand. Following gel elecrophoresis of PCR products, band intensities were quantitated using densitometry. Experiments were conducted in biological triplicate and the mean enrichment between target and control is indicated by the grey bars. A) The black bars at the left of the figure are the enrichments obtained using conventional ChIP in EBV-infected Ad/AH cells using an anti-EBNA1 antibody versus isotype control for the known EBNA1-binding dyad symmetry region within ori-P of EBV (EBV-DS) and for the c-jun promoter region (c-jun). Primer sets for promoter regions were within 1Kb upstream of the transcriptional start site. 5S RNA, tRNA^Tyr ^and 7SL RNA refer to intragenic promoter regions for these pol III-transcribed genes whilst 7SL UR refers to the upstream regulatory region of 7SL RNA. B) Enrichments for ATF2 and EBER promoter regions obtained using HaloEBNA1 pull-down in Ad/AH-EBERs cells.

Previously, we have used ChIP assays to show that EBNA1 is present at the promoters of several AP-1 subunits, including ATF-2, that are transcriptionally induced by EBNA1 in epithelial cells [31]. Here, we use a variant (HaloChIP) of conventional ChIP to demonstrate that EBNA1 is present at the promoters of genes encoding EBER-associated transcription factors that are induced in EBNA1-expressing cells. Using a transiently-expressed fusion protein construct (HaloEBNA1), we were able specifically to pull-down EBNA1 and associated DNA in an antibody-independent manner. The tagged version of EBNA1 is bound covalently by a resin; a specific interaction that can be prevented by a blocking ligand. We first validated the HaloChIP method by performing a comparison of HaloChIP with conventional ChIP in EBV-infected Ad/AH cells using an anti-EBNA1 antibody that, in such assays [39] had already been demonstrated to be competent to enrich the known EBNA1-binding DS region of EBV DNA and the cellular c-jun promoter region [31]. The enrichment obtained using the two methods was very similar (compare the c-jun data in Figure 7A). Using HaloChIP in Ad/AH cells, we were able to confirm that the EBNA1-fusion protein is present at the ATF-2 and c-Myc promoters through observation of a substantial enrichment of promoter DNA in experimental samples, compared with control samples (Figure 7A). Furthermore, we demonstrate that EBNA1 is present at the promoters of all TFIIIC subunit genes except TFIIIC63. This is consistent with the fact that TFIIIC63 was unique amongst the TFIIIC subunit genes in not responding to EBNA1 in Ad/AH cells. We also did not observe any enrichment of promoter DNA for TFIIIB genes or indeed cellular pol III-transcribed genes encoding tRNA^Tyr^, 5S RNA and 7SL RNA. The GAPDH promoter, included as a negative control, was not enriched in experimental samples compared with controls.

Although EBNA1 was not associated with the promoters of cellular pol III-transcribed genes it was of interest to determine whether the enhancement of EBER expression in EBNA1-expressing cells could be attributed to EBNA1 binding to the viral EBER promoters. Since, in the EBV genome, the EBER genes are located in close proximity to the EBNA1-binding OriP region [40], this study had to be performed in the Ad/AH-EBERs cell line, (see Methods) in which the EBERs are stably expressed from their native promoter elements in the absence of OriP which would have hampered the interpretation of ChIP assay data. HaloChIP assays performed in this cell line revealed a level of enrichment of the ATF2 promoter similar to that observed in Ad/AH-EBV cells. In contrast, as with the cellular pol III-transcribed genes, there was no enrichment of promoter DNA for the EBERs (Figure 7A, 7B).

### Transient expression of EBNA1 increases levels of EBER expression

**Figure 8:**
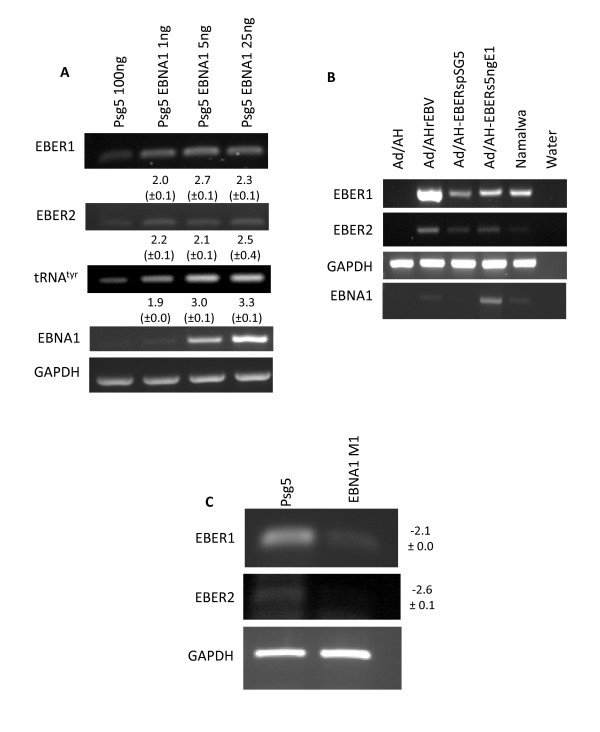
**EBNA1 expression leads to increased levels of EBER expression**. RT-PCR assay for (a) tRNA^Tyr ^and EBER expression in response to transient expression of increasing amounts of EBNA1 in the EBER-expressing cell line Ad/AH-EBERs. The experiments were conducted in biological triplicate. Numbers below the bands in the EBER1, EBER2 and tRNA rows represent the mean fold increase (± SD) in expression between the EBNA1-positive cells and the control as assessed by densitometry. (b) RT-PCR comparing EBER and EBNA1 expression in AdAH cell lines with levels seen in the Burkitt's lymphoma-derived cell line, Namalwa. (c) EBER expression in Ad/AH-rEBV cells transiently transfected with dominant negative EBNA1 vector or pSG5 control plasmid. Numbers to the right indicate the mean fold decrease (± SD) of EBER expression in triplicate experiments.

The data presented above confirm that EBNA1 is able to induce several factors involved in the regulation of EBER expression (TFIIIC, ATF-2 and c-Myc). However, these experiments fail to illustrate definitively that EBNA1 increases EBER expression. To address this, an EBNA1-expressing plasmid (pSG5 EBNA1) was titrated into the Ad/AH-EBERs cell line and the effect on EBER expression was assessed by RT-PCR (Figure 8a). Small amounts (down to 1ng) of EBNA1-encoding plasmid DNA were sufficient to increase levels of expression of both EBER1 and EBER2. This increase can be attributed to changes in shared components of the pol III machinery, since levels of tRNA transcripts are also increased. It is noteworthy that even the lowest level of EBNA1 is sufficient to elicit the responses and that overexpression is not required. Levels of EBER expression are shown to be comparable to those seen in the Burkitt's Lymphoma (BL) cell line Namalwa (Figure 8b).

### A dominant negative EBNA1 (dnEBNA1) reduces EBER expression in EBV-infected Ad/AH cells

As a converse of the experiments described above, ablation of EBNA1 function through introduction and expression of dnEBNA1 (EBNA1 M1) in EBV-infected Ad/AH cells resulted in a robust decrease in levels of both EBER 1 and EBER 2 expression (Figure 8c).

### Knock-down of EBER-specific transcription factors influenced by EBNA1 reduces EBER expression

**Figure 9:**
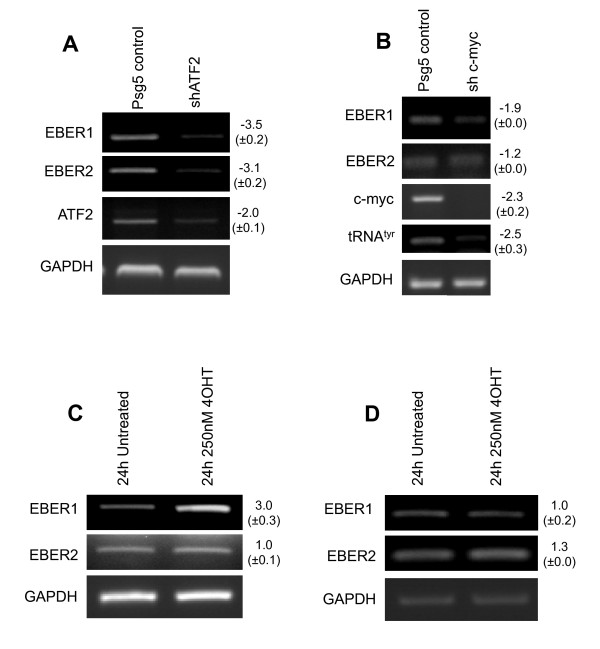
**ATF-2 and c-Myc induction is significant in regulating EBER expression**. RT-PCR was used to assay (a) EBER expression following transient shRNA knock-down of ATF-2; (b) EBER expression following transient shRNA knock-down of c-Myc; (c) EBER expression following tamoxifen induction of c-MycER in Ad/AH-EBERs cells; (d) EBER expression following tamoxifen induction of c-MycER in Ad/AH ΔX EBER cells with the c-Myc binding site (X-box upstream of EBER1) excised. Numbers to the right of the gel images indicate the mean fold change (± SD) of expression in triplicate experiments.

To confirm the influence of EBNA1-induced transcription factors ATF-2 and c-Myc on EBER expression, we used shRNA-mediated knock-down of these factors in EBV-infected Ad/AH cells and examined, using RT-PCR, the effect on EBER expression. shRNA targeting ATF-2 robustly reduced ATF-2 expression and, as a result, expression of both EBER1 and EBER2 was significantly diminished (Figure 9a).

shRNA targeting c-Myc also successfully reduced levels of c-Myc transcription in EBV-infected Ad/AH cells, and was associated with a diminished level of EBER1 but no apparent reduction in that of EBER2 (Figure 9b). This is consistent with the observation that only the EBER1 gene contains an E-Box (c-Myc binding site) upstream of its transcriptional start site [21]. Previously, c-Myc has been shown to increase levels of pol III transcription directly by interacting with TFIIIB subunits [41]. Consistent with this, RT-PCR conducted for endogenous pol III targets (Figure 9b) showed that shRNA-mediated depletion of c-Myc reduced expression of cellular pol III products (data shown for tRNA^Tyr^).

### Induction of a functional c-Myc influences EBER expression

To assess further the contribution of EBNA1's ability to enhance the transcriptional activation potential of c-Myc leading to increased EBER expression, an Ad/AH cell line was generated which stably expressed both c-MycER and the EBERs (including their upstream transcriptional regulatory regions). Following functional induction of c-Myc, levels of EBER expression were assessed (Figure 9c). After 24 hours of induction, a clear increase in the level of EBER1 was observed, but only a very modest increase in that of EBER2. This result is consistent with the shRNA data and supports the idea of the E-boxes upstream of EBER1 being important in terms of transcriptional regulation of the EBERs. To confirm this, an Ad/AH cell line was generated which stably carried both c-MycER and the EBERs, including their upstream transcriptional regulatory regions (TATA, SP1 and ATF sites), but with a ~230 bp region containing the E-boxes excised (see Materials and Methods). Again, levels of EBER expression were assessed by RT-PCR following induction (Figure 9d). Levels of EBER1 or EBER2 expression were not significantly increased upon functional induction of c-Myc in this cell line, again indicating that c-Myc influences EBER transcription via the upstream E-boxes.

### TFIIIC102 is upregulated in EBV-positive NPC tumour cells

**Figure 10:**
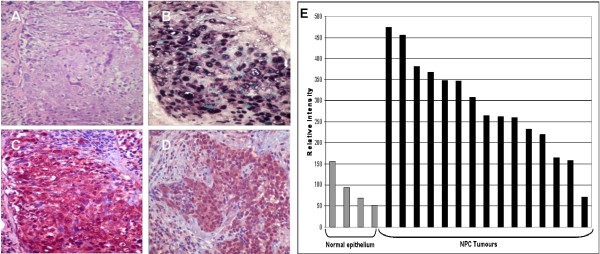
**TFIIIC-102 is upregulated in EBV-positive NPC tumour cells**. Serial sections of an NPC biopsy were (A) stained with haematoxylin and eosin; (B) shown to contain strongly EBER-positive tumour cells by *in situ *hybridisation; (C) strongly positive for TFIIIC-102 compared with the surrounding stromal, non-tumour cells. (D) Shows similar staining in a different biopsy. (E) Levels of mRNA for TFIIIC-102 in tumour tissue compared with normal epithelium.

The potential relevance *in vivo *of our observations of EBNA1-induced induction of TFIIIC102 was addressed by immunohistochemical staining of EBV-positive NPC tumour biopsies (Figure 10a - d). In 18 out of 19 NPC biopsies, nuclear staining for TFIIIC102 appeared significantly stronger in tumour cells than in infiltrating non-tumour cells. Additionally, microarray analysis of RNA extracted from 15 samples of microdissected EBV-positive NPC tumour cells compared with 4 samples from normal epithelial cells, revealed consistent induction of TFIIIC102 mRNA in the tumours (Figure 10e). Expression of other TFIIIC subunits was variable and so low in many samples as to be called "absent" by the analysis software.

## Discussion

It has long been established that high levels of EBER expression are a feature of cells latently infected with EBV and in cells of EBV-associated malignancies [18]. The findings presented here provide evidence that EBNA1 plays a significant role in the regulation of EBER expression in epithelial cells. We show that EBNA1, like the adenovirus E1A protein [24] and the T-antigens of polyomaviruses [22,25], increases cellular pol III transcription and induces expression of specific TFIIIC subunits. In addition, other EBER-associated transcription factors, ATF-2 and c-Myc, are shown to be transcriptionally upregulated, with EBNA1 being present at the promoters both of these genes and those of TFIIIC subunits. EBER expression is shown to be adversely affected by reduction of ATF-2 and c-Myc, highlighting the significance of EBNA1's ability to induce these transcription factors. Furthermore, EBNA1 was shown to enhance levels of EBER expression, illustrating its importance in the characteristic abundant expression of EBERs that is associated with EBV latent infection.

Our findings build upon our previous study [29] which demonstrated that cellular pol III transcription and TFIIIC are induced in EBV-infected epithelial cells. The significance of increased levels of cellular pol III transcription is not restricted to increased levels of viral transcripts such as the EBERs. It is well-established that pol III transcription is often deregulated in a wide range of malignant tumours and transformed cells [38,42-44]. Furthermore, recent data suggest that increased pol III transcription is necessary or even sufficient to promote proliferation and oncogenic transformation in some contexts [38,45]. Our *in vivo *findings support the idea of increased pol III transcription in malignant tumours, where induction of pol III-specific transcription factors was observed in NPC tumour samples at both the mRNA and protein level. Given our evidence for induction of TFIIIC subunits in EBNA1-expressing epithelial cells, and the well-established association between EBV and NPC, it is tempting to speculate that increased TFIIIC subunit levels in NPC tumour cells could be mediated, at least in part, by EBNA1.

Our data also address EBNA1's role in upregulation of the transcription factor ATF-2 that has previously been shown to be important for expression of EBERs [20,46,47]. Earlier studies are conflicting in their descriptions of the effect of EBV on ATF-2. Initial studies revealed that EBV triggers hyperphosphorylation of ATF-2 leading to increased activity [1,48-51], with increased levels of mono-phosphorylated ATF-2 being observed in the absence of any increase in total levels of ATF-2. However, recent data from our laboratory [31] revealed that EBNA1 increases the level of ATF-2 transcription and that it is present at the promoter of the gene. Using shRNA, in the current work we show that by reducing ATF-2 transcripts, levels of both EBERs are reduced significantly in EBV-infected Ad/AH cells (Figure 9a). This supports the previous data suggesting the importance of ATF-2 and ATF-2 binding sites upstream of EBER genes to the expression of EBER transcripts. Further, our data illustrating increased levels of both total and phosphorylated (activated) ATF-2 suggest that EBNA1 acts to increase EBER expression both through induction of ATF-2 transcription and the resultant increase in levels of dual-phosphorylated and activated ATF-2.

Through its interaction with TFIIIB, c-Myc can directly activate many cellular pol III-transcribed genes [41]. This is consistent with the observed decrease in tRNA expression that accompanies c-Myc RNAi (Figure 9b). However, EBER2 appears to be an exception, as it shows little response to RNAi or overexpression of c-Myc, at least in Ad/AH cells (Figure 9b-d). This may reflect the unusual promoter arrangement and factor requirements of EBER genes, which differ from most cellular pol III promoters [29]. In contrast to EBER2, the upstream regulatory region of EBER1 contains two adjacent E-box DNA sequences that can be bound directly by c-Myc [21]. By excising a short region containing these motifs, we revealed its requirement for induction by activated MycER (Figure 9d). This contrasts with a previous report that this upstream region has little importance for EBER1 expression in 293 cells [47]. Differences between cell types or the fact that the earlier work [47] used a transient transfection assay may be responsible for the discrepancy. Our data suggest that the EBER genes show differential responsiveness to c-Myc due to E-box DNA motifs upstream of the EBER1 promoter. Our results showing upregulation of c-Myc in response to EBNA1 are consistent with the findings of an earlier study [35]. In contrast, another study [52] found that expression of EBNA1 led to a reduction in the level of c-Myc. The authors of this latter study [52] propose that cell- and tissue-specific effects may be responsible for a variety of discordant observations.

Since EBNA1 induces EBER-associated cellular transcription factors, associates with their promoters (Figure 7, [31]) and has been shown to bind to multiple sites within the human genome [53], we addressed the possibility of EBNA1 association with multiple EBER-related transcription factors using chromatin pull-down experiments and found EBNA1 to be associated with the promoters of these factors. The presence of EBNA1 at these promoters affords a potential mechanistic insight into EBNA1's mode of action in enhancing expression of EBERs. The nature of the interaction between EBNA1 and upregulated genes is, at present, unknown. However, whilst the EBNA1-binding motif defined in the EBV genome was absent from the promoters that show EBNA1 association, two recent studies have reported that EBNA1 can bind to cellular genomic DNA [52,53]. Further, Canaan et al. [52] identified several, hitherto uncharacterised EBNA1 DNA binding motifs present in the promoter regions of cellular genes. We found that two of these, WTACTTT and GRAGTATY were present within 1 kb of the transcriptional start sites of the EBNA1-associated ATF2, c-Myc, TFIIIC220 and TFIIIC90 promoters (compatible with the resolution of detection in our ChIP assays). In contrast, these motifs are not present in the promoters of c-Jun or the other TFIIIC subunits whose expression was found to be up-regulated in EBNA1 expressing cells and for which HaloChIP assays indicated EBNA1 binding at their promoters. It is possible that EBNA1's interaction with cellular promoters may be direct in some instances yet indirect in others. Elucidating the mechanisms by which EBNA1 associates with different cellular promoters warrants further investigation.

Although our data indicate that EBNA1, through a variety of mechanisms can induce EBER expression, we note that the levels of expression in this model system are low when compared with those in Ad/AH cells stably infected with EBV. This may be attributable to the EBER expression plasmids in the model system being maintained in an integrated form. In this context it is notable that the Namalwa cell line, in which EBV is known to be integrated at low copy number [54], also expresses the EBERs at low levels [18,55], similar to those seen in our model system (Figure 8b). In the context of whole virus infection, the EBV genome is usually maintained as a multi-copy episome. In this scenario, transcription factor binding access and kinetics may be very different, given that the EBER genes are adjacent to *oriP*, within the region of unusual chromatin structure [47]. The *oriP *region (absent from the Ad/AH-EBER cell line) is also known to be a transcriptional enhancer region [32,34] and has been previously demonstrated to increase EBER expression by 2- to 4-fold [47]. The number of copies of the EBER genes integrated into the Ad/AH-EBER cell line generated is unknown, though it is unlikely to reach the number of viral episomes carried by EBV-infected Ad/AH cells. Further, it is of note that EBNA1 does not seem to be capable of inducing TFIIIB subunit expression. We anticipate that pol III-transcribed genes (such as the EBERs) would be even more highly expressed in cells in which TFIIIB was induced in addition to TFIIIC, an environment which is observed in EBV-infected cells [29].

## Conclusions

EBNA1 has previously been shown to possess transcriptional-regulatory properties for a number of pol II-dependent EBV latent genes. The data presented here extend the repertoire of its stimulatory mechanisms to include the pol III-transcribed EBER genes. This could have a major impact, given reports of EBER oncogenicity. Cellular pol III products are also induced by EBNA1. Its capacity to produce these effects can be explained by an ability to bind and activate expression of genes encoding ATF-2, c-Myc and TFIIIC, transcription factors shared by the viral and cellular pol III templates.

## Methods

### Plasmids

The EBNA1 expression plasmid pSG5 EBNA1 [33], c-Myc reporter pX-CMVp-Luc [21] and dominant-negative EBNA1 expression vector EBNA1-M1 [56] were as described. A plasmid containing a functionally-inducible c-Myc gene (a fusion between the hormone binding domain of the oestrogen receptor and c-Myc) [57] was obtained from C. Tselepis. An EcoRI fragment containing the fusion (c-MycER) was ligated into pcDNA3.1/zeo (Invitrogen). The pUC19 EBERs plasmid was generated by inserting the 1 kb SacI - EcoRI subfragment from the EcoRI J fragment of the EBV genome into the corresponding sites of pUC19, with a puromycin resistance cassette [58] being inserted at the SalI site. For generation of pUC19 Puro EBERs ΔX, the X-box c-Myc binding site was excised from pUC19 EBERs plasmid using SacI and PmlI, excising bases -352 to -125 relative to the EBER1 transcriptional start site. A control Renilla luciferase plasmid (pRL-TK) was from Promega.

### Cell lines and tissue culture

Ad/AH (a human adenocarcinoma cell line derived from the nasopharynx), HONE-1 (an EBV-negative NPC cell line) and AGS (human gastric carcinoma-derived) cell lines were cultured in RPMI 1640 medium supplemented with 10% fetal calf serum (FCS), 2 mM L-glutamine and 1% penicillin-streptomycin solution (Sigma-Aldrich). HONE-1 and AGS cells stably expressing EBNA1 and the Ad/AH line stably infected with a recombinant EBV were described previously [35]. An Ad/AH cell line that stably expresses physiological levels of EBNA1 (Figure 2) was derived as described previously [35].

Ad/AH-Neo and -EBNA1 cells stably expressing the c-MycER fusion protein were generated by transfection of cells with c-MycER plasmid followed by drug selection with Zeocin (Invitrogen). Ad/AH-EBERs cell lines (and derivatives) were generated by stable transfection of pUC19 EBERs plasmid, followed by puromycin drug selection of polyclonal EBER-positive cell populations.

### Luciferase assays and transient transfections

Dual luciferase reporter assays were performed according to the manufacturer's instructions (Promega) with cells cultured in RPMI 1640 supplemented with 0.5% FCS, 2 mM L-glutamine, and 1% penicillin-streptomycin solution. Cells were transfected with plasmids using Lipofectamine (Invitrogen) according to the manufacturer's instructions. All assays were carried out in biological and technical triplicate and are represented as the mean of three independent experiments.

Cells were subjected to shRNA directed against c-Myc (pGIPZ 152051, OpenBiosystems) and ATF-2 (pLKO.1 13713, OpenBiosystems) using transfection techniques previously outlined. RNA was extracted from cells 72 h post-transfection.

### ChIP assays

ChIP assays were performed as described previously [31] using an EBNA1 antibody (chEBNA1) and conditions described by Chau & Lieberman (2004) [39]. A rabbit isotype control antibody was obtained from Santa Cruz. Precipitated target sequences were identified by PCR and quantitated by densitometry as described below.

### HaloCHIP assays

HaloCHIP assays were performed according to the manufacturer's instructions (Promega, UK). HaloEBNA1 fusion protein-encoding plasmid was generated using PCR based cloning techniques, with pseudo-wild type EBNA1 (with deleted Gly/Ala repeat region) fused to HaloTag protein (N-terminal) in pFC14K-CMV backbone plasmid (Promega UK). 0.5 μg HaloEBNA1 plasmid was transfected per 10 cm^2 ^dish. Dishes were formaldehyde cross-linked 24 h post transfection before manufacturer's protocols were followed precisely.

PCR was performed using GoTaq Green Polymerase Mastermix (Promega UK). Primer sets for promoter regions were designed with the aid of literature searches for known promoter regions. All primer sets were based upon regions within 1 Kb upstream of transcriptional start sites. Exceptionally, primer sets for cellular pol III-transcribed genes tRNA^Tyr ^and 5S RNA were based, due to their intragenic promoters, on regions internal to the gene. 7SL RNA's promoter regions are both intragenic and upstream of the transcriptional start site therefore primer sets for both regions were used. Primer sets are listed in Table 1. The linear range of each PCR reaction was determined empirically through semi-quantitative PCR to determine experimental cycle numbers.

**Table 1:** PCR primers used in ChIP and HaloCHIP experiments.

Gene (promoter region)	Primer oligonucleotides (5'-3')
TFIIIC220*	Forward: GTTTGCAGTTCCCCTGGTTAC Reverse: CTTCGTCCAACAACGACTCC

TFIIIC110*	Forward: TCTCCCCTTTTTGACACTGC Reverse: AGGGGGAGGAGTAATTGTGG

TFIIIC102	Forward: CGGTTCCTTGCTCTTGCT Reverse: GCATCGCCTCACTTTCTT

TFIIIC90	Forward: GCGGGTAGGGACAAGACT Reverse: CCCAGACAGGCTTTAGTT

TFIIIC63	Forward: GCCAACACCGACAGAATA Reverse: CGGAGGATAATAAGACACAAA

5S RNA	Forward: TTTACGGCCACACCACCCTG Reverse: AAAGCCTTCAGCACCCTGTA

tRNA^tyr^	Forward: CCTTCGATAGCTCAGCTGGTAGAGCGGAGG Reverse: CGGAATTGAACCAGCGACCTAAGGATGTCC

7SL RNA (gene)	Forward: GTGTCCGCACTAAGTTCGGCATCAATATGG Reverse: TATTCACAGGCGCGATCCCACTACTGATC

7SL RNA^†^ (upstream promoter)	Forward: CCGTGGCCTCCTCTACTTG Reverse: TTTACCTCGTTGCACTGCTG

Brf1	Forward: GCAAGGAGGTCAGGCACT Reverse: CCTTCCACGGCTACCTCT

Bdp1	Forward: GTTTCTTCACACCAGCATT Reverse: GCTACTGAGACTGGGTTA

TBP	Forward: CGCCCCTCCTTACCTAT Reverse: CAATCTGTTACCTGGGTC

ATF-2	Forward: CCCAAACCTCACCTAACCCGAAG Reverse: CTCGGGCGCTCATGATTGGACAA

c-myc	Forward: TCCTCTCTCGCTAATCTCCGC Reverse: CCCTCCGTTCTTTTTCCCG

c-jun	Forward: CCCACAAGTGGGGAAACAACAA Reverse: GCTACCAGTCAACCCCTAAAAATA

GAPDH	Forward: TACTAGCGGTTTTACGGGCG Reverse: TCGAACAGGAGGAGCAGAGAGCGA

EBV-DS	Forward: CCGTGACAGCTCATGGGGTGGGAGAT Reverse: CAATCAGAGGGGCCTGTGTAGCTACCG

EBERs	Forward: CCGCCTACACACCAACTAT Reverse: GGGATTAGAGAATCCTGACTT

* Primer sets previously described [29]. ^†^Primer set previously described [62].

### RT-PCR, immunoblotting, immunofluorescence and immunohistochemistry

RNA for RT-PCR was reverse transcribed with Superscript III, (Invitrogen) following the manufacturer's protocol, using random primers (Promega UK). Resultant cDNA was then used for PCR reactions as above. Primers are listed in Table 2. Standard immunoblotting procedures [35] were used to detect total ATF-2, (mouse MAB1536, 1 mg/ml, Santa Cruz), mono- (Thr69) and dual-phosphorylated (Thr69/71) ATF-2 (mouse MAB1536 1 mg/ml, Cell Signaling Technology), EBNA1 (human serum, 1:500), actin (mouse AC-38, 1:20 000, Sigma). Immunoblotting for TFIIIC subunits was performed as described previously [29]. Immunohistochemical staining and EBER *in situ *hybridisation were performed on biopsies used in previous studies [59] and were conducted as described therein. For immunohistochemistry, antibody 3238 to TFIIIC-102 [29] was used at a dilution of 1:100 following standard techniques. Immunofluorescence was carried out on Ad/AH-Neo and -EBNA1 cells using standard procedures [35] with total ATF-2 antibody (mouse MAB1536, 0.5 mg/ml, Santa Cruz).

**Table 2:** RT-PCR primers.

Gene	Primer oligonucleotides (5'-3')
ATF-2	Forward: CACACAACTCCACAGACCCAAA Reverse: GGAGCCATAACGATCTGTGAAA

c-Myc	Forward: AACCAGAGTTTCATCTGCGACCCG Reverse: TTGTGCTGATGTGTGGAGACGTGG

5S RNA*	Forward: TTTACGGCCACACCACCCTG Reverse: AAAGCCTTCAGCACCCTGTA

tRNA^tyr^*	Forward: CCTTCGATAGCTCAGCTGGTAGAGCGGAGG Reverse: CGGAATTGAACCAGCGACCTAAGGATGTCC

7SL RNA *	Forward: GTGTCCGCACTAAGTTCGGCATCAATATGG Reverse: TATTCACAGGCGCGATCCCACTACTGATC

TFIIIC 220*	Forward: TCCAGCGAGACCGTCACACC Reverse: GGATTGAGTGTTGCTGGGCT

TFIIIC 110*	Forward: CCAGAAGGGGTCTCAAAGTCC Reverse: CTTTCTTCAGAGATGTCAAAGG

TFIIIC 102*	Forward: GCAGAAGTAACATCATTGGC Reverse: CCTACTAATGTCCGTTATCTGTGG

TFIIIC 90*	Forward: AAACAGAAGTTGCTGAGTGC Reverse: ATGGTCAGGCGATTGTCC

TFIIIC 63*	Forward: ATGGCTTGAAGTCCTCCTCCTCC Reverse: CCGAGATGTTCTACCAGTTATGCG

Bdp1*	Forward: GCTGATAGAGATACTCCTC Reverse: CCAGAGACAAGAATCTTCTC

Brf1*	Forward: AAATTCTGTGAGCCTCTTCCGTAGTG Reverse: AGACCCATGCTTGTACATTCCACG

TBP*	Forward: GCCAGAGTTATTTCCTGGTTT Reverse: CCCAGATAGCAGCACGGTAT

GAPDH	Forward: GCCTCCTGCACCACCAACTG Reverse: CGACGCCTGCTTCACCACCTTCT

EBER1	Forward: AGGACCTACGCTGCCCTAGA Reverse: AAAACATGCGGACCACCAGC

EBER2	Forward: GCCGTTGCCCTAGTGGTTT Reverse: GGGATTAGAGAATCCTGACTT

EBNA1	Forward: CCGCAGATGACCCAGGAGAA Reverse: TGGAAACCAGGGAGGCAAAT

* Primer sets previously described [29].

### Densitometry

Densitometry was conducted on UV transilluminator images using ImageJ 1.37 V (http://rsbweb.nih.gov/ij/). Bands were analysed in technical triplicate, normalising against background image levels and, if appropriate, internal controls. In all instances, densitometry was conducted in biological triplicate.

### Quantitative RT-PCR (RT-qPCR)

RT-qPCR for cellular genes was performed using ready synthesised Taqman primer and probe (FAM labelled) mixes purchased from Applied Biosystems (see Table 3). Each multiplexed RT-qPCR reaction consisted of 5 μl (50 ng) of cDNA, 1 μl of ABI test primers and probe mix, 0.5 μl of huGAPDH primer and probe (VIC labelled) mix, 10 μl of 2× sensimix (Quantace) and DEPC water to 20 μl. Reactions were performed in biological and technical triplicate and analysed on an ABI 7500 Fast Real-time PCR machine. Data were analysed using the 2^-ddCt ^method [60].

**Table 3:** Applied Biosystems q-PCR assays.

Gene	ABI QPCR Primer/Probe Reference Number
TFIIIC220	Hs01121460_m1

TFIIIC110	Hs01086775_m1

TFIIIC102	Hs01066489_m1

TFIIIC90	Hs01034271_m1

TFIIIC63	Hs01030747_m1

BDP1	Hs00372575_m1

BRF1	Hs00377388_m1

TBP	Hs00920494_m1

### Microarray analysis of nasopharyngeal carcinoma (NPC) tumours

Full details of the protocol will be presented elsewhere (C. Hu et al., in preparation). Briefly, normal epithelial cells and EBV-positive NPC tumour cells were isolated by laser-capture microdissection from frozen biopsies. Total cellular RNA was extracted using a Qiagen RNEasy kit followed by amplification, labelling and hybridisation to Affymetrix U133 Plus2 arrays essentially as previously described [61]. After scanning, intensity values were analysed using GCOS software (Affymetrix).

## Competing interests

The authors declare that they have no competing interests.

## Authors' contributions

TJO participated in the design and interpretation of the study, carried out the majority of the experimental work and helped to draft the manuscript. JDO participated in the design and interpretation of the study, carried out some HaloChIP work and helped to draft the manuscript. CWD participated in the design and interpretation of the study. CH performed microarray work. XC and YY collected biopsies, performed tumour diagnosis and carried out immunohistochemical staining. VHJW derived cell lines and helped to draft the manuscript. LEM performed western blotting. RJW participated in the interpretation of the study and helped to draft the manuscript. LSY participated in the design and interpretation of the study. JRA participated in the design and interpretation of the study, carried out microarray work and helped to draft the manuscript. All authors read and approved the final manuscript.
